# Building capacity for evidence generation, synthesis and implementation to improve the care of mothers and babies in South East Asia: methods and design of the SEA-ORCHID Project using a logical framework approach

**DOI:** 10.1186/1471-2288-10-61

**Published:** 2010-07-01

**Authors:** Steve McDonald, Tari Turner, Catherine Chamberlain, Pisake Lumbiganon, Jadsada Thinkhamrop, Mario R Festin, Jacqueline J Ho, Hakimi Mohammad, David J Henderson-Smart, Jacki Short, Caroline A Crowther, Ruth Martis, Sally Green

**Affiliations:** 1 Australasian Cochrane Centre, Monash University, Melbourne, VIC 3168, Australia; 2 Department of Obstetrics and Gynaecology, Khon Kaen University, Khon Kaen 40002, Thailand; 3 Department of Obstetrics and Gynecology, College of Medicine, University of the Philippines Manila, Manila 1000, Philippines; 4 Department of Paediatrics, Penang Medical College, Penang 10450, Malaysia; 5 Department of Obstetrics and Gynecology, Gadjah Mada University, Yogyakarta 55281, Indonesia; 6 NSW Centre for Perinatal Health Services Research, Queen Elizabeth II Research Institute, University of Sydney, NSW 2006, Australia; 7 Discipline of Obstetrics and Gynaecology, University of Adelaide, SA 5006, Australia

## Abstract

**Background:**

Rates of maternal and perinatal mortality remain high in developing countries despite the existence of effective interventions. Efforts to strengthen evidence-based approaches to improve health in these settings are partly hindered by restricted access to the best available evidence, limited training in evidence-based practice and concerns about the relevance of existing evidence. South East Asia - Optimising Reproductive and Child Health in Developing Countries (SEA-ORCHID) was a five-year project that aimed to determine whether a multifaceted intervention designed to strengthen the capacity for research synthesis, evidence-based care and knowledge implementation improved clinical practice and led to better health outcomes for mothers and babies. This paper describes the development and design of the SEA-ORCHID intervention plan using a logical framework approach.

**Methods:**

SEA-ORCHID used a before-and-after design to evaluate the impact of a multifaceted tailored intervention at nine sites across Thailand, Malaysia, Philippines and Indonesia, supported by three centres in Australia. We used a logical framework approach to systematically prepare and summarise the project plan in a clear and logical way. The development and design of the SEA-ORCHID project was based around the three components of a logical framework (problem analysis, project plan and evaluation strategy).

**Results:**

The SEA-ORCHID logical framework defined the project's goal and purpose (*To improve the health of mothers and babies in South East Asia *and *To improve clinical practice in reproductive health in South East Asia*), and outlined a series of project objectives and activities designed to achieve these. The logical framework also established outcome and process measures appropriate to each level of the project plan, and guided project work in each of the participating countries and hospitals.

**Conclusions:**

Development of a logical framework in the SEA-ORCHID project enabled a reasoned, logical approach to the project design that ensured the project activities would achieve the desired outcomes and that the evaluation plan would assess both the process and outcome of the project. The logical framework was also valuable over the course of the project to facilitate communication, assess progress and build a shared understanding of the project activities, purpose and goal.

## Background

Poor health and pregnancy care leads to over half a million maternal deaths and almost eight million perinatal deaths each year. For every woman who dies, about 20 more suffer injuries, infection and disabilities [1]. The burden of death and injury falls disproportionately on low and middle income countries, as is indicated by the lifetime risk of maternal death in South East Asia which is one in 130 compared to one in 7,300 in developed regions [2]. Importantly, much of this burden could be prevented if interventions which have been demonstrated by research to be effective and feasible were implemented at scale [3].

Implementation of the results of research into practice has been identified by the WHO as the most significant challenge to health care [4]. An overview of approaches to bridging the gap between research and practice has highlighted the lack of primary research about what interventions are effective in increasing use of research in health care in low-income settings [5]. In part this reflects the low priority afforded to health services research in these settings [6]. This is a concern given that increasing the generation and use of research in health care are essential steps in improving health systems and achieving better health, particularly in low-income settings [7-9].

Ensuring access to scientifically valid and up-to-date information is a prerequisite to use of research in guiding health care, or 'evidence-based practice'. In spite of the profusion of health information available in print and electronic media (and several international initiatives to promote access), healthcare workers in low-income settings are still disadvantaged when it comes to accessing reliable information on effective care [10,11]. Organisational, financial and technical barriers to accessing research (including lack of computers and internet access, limited scientific literacy, lack of availability of research, inappropriate format of research publications, etc.) and concerns about relevance to local settings are compounded by a lack of skills among practitioners and other stakeholders to appraise and interpret research findings [12].

The South East Asia - Optimising Reproductive and Child Health in Developing Countries (SEA-ORCHID) project aimed to address some of these issues. This five-year project (2004-08) was funded through the International Collaborative Research Grants Scheme, a partnership between the UK-based Wellcome Trust and Australia's National Health and Medical Research Council. The scheme was designed to foster collaborative research between countries in South East Asia and Australia by funding research into major health issues affecting developing countries and developing research capacity.

The objective of SEA-ORCHID was to evaluate whether a intervention designed to strengthen the capacity for research generation, synthesis, and use, improved perinatal practice and led to better health outcomes for mothers and babies.

The SEA-ORCHID study protocol reporting the overall research design and project plan has been published previously [13]. The protocol outlined the core groups targeted by the intervention and key intervention activities. Planning and designing the intervention was undertaken in two stages: development of a project-wide logical framework before the intervention phase; and a site-specific action research-based operationalisation of this framework undertaken during the intervention phase. In this paper, we describe the methods and results of the first stage.

## Methods

**Figure 1:**
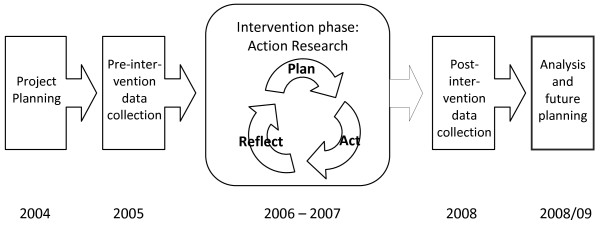
SEA-ORCHID Project schema

The SEA-ORCHID project was a pragmatic study that used an uncontrolled before-and-after design to evaluate the impact of a multifaceted tailored educational intervention, implemented within an action research framework (Figure 1). The intervention consisted of a wide range of components and strategies, based on sound educational principles, tailored to meet the needs, priorities and resources of each of the participating hospitals.

The project was approved by the ethics committee relevant to each participating hospital and by the ethics committee of the administering institution in Australia (University of Sydney).

### Logical Framework Approach

In complex projects that have broad goals, a logical framework approach can help summarise and describe the multiple strands of the project clearly and systematically, and clarify the core goals, broad objectives, activities and expected outcomes [14]. There are three main components of a logical framework: project rationale (analysis of main problems and their causes); project plan (goal, objectives, activities, outputs) and evaluation and measurement strategy. The logical framework tool helps develop and demonstrate the *logic *of the project in two dimensions. The first, vertical dimension, is whether the activities of the project are likely to achieve the objectives and whether these objectives are likely to achieve the broader goals. The second, horizontal dimension, is whether the evaluation plan will measure the project's progress (process measures) and whether the progress of the project will in turn address the identified problems (outcome measures).

### Development of the logical framework

The SEA-ORCHID project coordinator (SM) and one of the Australian-based project staff (CC) managed the development of the logical framework, drawing on information from several sources, along with contributions from members of the SEA-ORCHID team.

### Information sources

The information sources used to develop the logical framework included the study protocol [13], a brief survey of perceived current practice, and in-depth discussions at investigator and project meetings (to capture the experiences and perspectives of the local decision-makers). The study protocol set the parameters of the project in terms of its overall aims and design.

At the start of the project planning phase, the SE Asian investigators completed an informal survey of current practice which provided a snap shot of the extent to which care was in line with research (as perceived by the investigators who were senior clinicians working in the participating sites) at different levels within the hospital system in each country (tertiary, regional, provincial and district). The survey also collected data on any complementary research activities that might inform or affect the intervention design, such as participation in the *WHO Reproductive Health Library *cluster randomised trial [15] (Additional file 1: SEA-ORCHID survey).

Discussions at the early investigator meetings were pivotal in articulating the key issues and beginning the process of translating these into objectives linked to activities. At the first full meeting of all the investigators and Australian-based educators in Adelaide in March 2005, several general principles were agreed with respect to intervention planning:

• sustainability and capacity-building should underpin all intervention strategies;

• interdisciplinary teamwork, including both clinical and non-clinical staff, such as librarians and biostatisticians, should be encouraged;

• SEA-ORCHID project baseline data on clinical practices should be used wherever possible to determine local priorities.

During the same meeting, each SE Asian investigator summarised the current situation at the project hospitals in their country in terms of awareness, acceptance and use of research in practice; outlined particular barriers and enablers that could affect the intervention; and suggested activities they considered priorities.

The information from these three sources served as the raw material for the logical framework.

### Contributors

The logical framework was developed with input from the seven chief investigators (one from each of the SE Asian countries, and three from Australia), the two project coordinators (from Australia and Thailand) and the Australian project staff.

The SE Asian investigators were senior obstetricians or neonatologists responsible for co-ordinating the project and determining both local and national priorities within the overall framework of the project.

**Figure 2:**
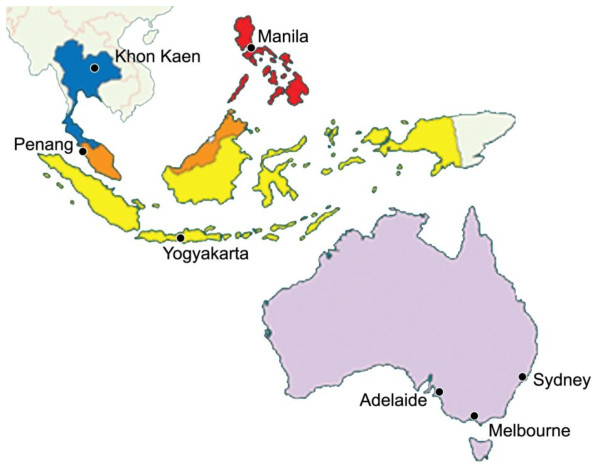
Location of SEA-ORCHID Project partners

The selection of investigators was determined on the basis of existing partnerships and networks within SE Asia and Australia (Figure 2). Professional links have been fostered over many years through regular meetings of Federation of Asia and Oceania Perinatal Societies and the Perinatal Society of Australia and New Zealand. The participating centres and their respective investigators in Thailand (Khon Kaen University, Khon Kaen), The Philippines (University of the Philippines, Manila) and Indonesia (Gadjah Mada University, Yogyakarta) collaborate on studies initiated by the World Health Organization and are also linked through INCLEN (International Clinical Epidemiology Network) and the Global Network for Perinatal and Reproductive Health. In Thailand, Khon Kaen University also serves as the co-ordinating base of the Thai Cochrane Network. The participating centre in Malaysia (initially at Ipoh Hospital and then Penang Medical College) is the focal point for initiatives to support and develop Cochrane activity.

The three Australian investigators were from centres that have strong links with The Cochrane Collaboration and international research networks for maternal and perinatal health. The Australian Research Centre for Health of Women and Babies (ARCH) at the University of Adelaide http://www.health.adelaide.edu.au/og/research/arch.html comprises several research divisions. The research synthesis division, where the regional co-ordinating unit for the Cochrane Pregnancy and Childbirth Group is based, also includes the ARCTURUS Clinical Trials division and the national coordinating office for the WOMBAT Collaboration http://www.wombatcollaboration.net and promotes and supports high quality randomised trials in maternal and perinatal health.

The Centre for Perinatal Health Services Research at the University of Sydney aims to improve the process and outcome of care for mothers and infants in New South Wales, nationally and internationally, through research and education promoting evidence-based practice http://www.psn.org.au/. The Centre is also the regional co-ordinating base for the Cochrane Neonatal Group. The third Australian site was at Monash University and houses the Australasian Cochrane Centre http://www.cochrane.org.au/, which is responsible for training and supporting review authors and fostering the development of Cochrane activity in Australasia and South East Asia.

The nine study sites (hospitals) were purposively selected to include tertiary referral hospitals (University and regional), provincial hospitals and district hospitals. All were public hospitals and ranged in size from a district hospital in Indonesia with 100 births a month to a tertiary referral hospital in the Philippines with up to 2500 births a month (Table 1). We selected hospitals in which the SEA-ORCHID team members were actively involved in providing clinical services to ensure that the project would be locally owned and that project development, implementation and evaluation would be participatory. The participatory approach to designing the intervention plan did not extend to formal engagement with local decision-makers or community members who were not actively involved in SEA-ORCHID.

**Table 1:** Hospitals in the SEA-ORCHID Project

Country	Hospital site	Type of hospital	Average births per month (in 2005)
**Indonesia**	Dr Sardjito Hospital, Yogyakarta	Tertiary	100
	
	Sleman District Hospital, Yogyakarta	District	100

**Malaysia**	Ipoh Hospital, Ipoh	Tertiary	750
	
	Kelantan Hospital, Kota Bharu	Tertiary	650

**Philippines**	Philippine General Hospital, Manila	Tertiary	500
	
	Jose Fabella Hospital, Manila	Tertiary	2500

**Thailand**	Srinagarind Hospital, Khon Kaen	Tertiary	200
	
	Khon Kaen Hospital, Khon Kaen	Regional	300
	
	Kalasin Hospital, Kalasin	Provincial	350

### Development process

Development of the logical framework included establishing a project rationale by defining and unpacking the central problem - the problem analysis - and developing a project goal that captured the overarching aim of the project in addressing the central problem.

The project planning component of the process sought to generate a range of possible activities and outputs designed to meet the objectives underpinning the project's overall aim. The third component of the logical framework - evaluation and measurement - outlined the process measures designed to monitor the project's progress and the outcome measures designed to assess whether the project was meeting its goal and purpose.

Refining the logical framework was a four-month, iterative process with drafts circulated among the project teams for feedback and comments. The final version of the logical framework was approved in June 2005.

## Results

### Project rationale

**Figure 3:**
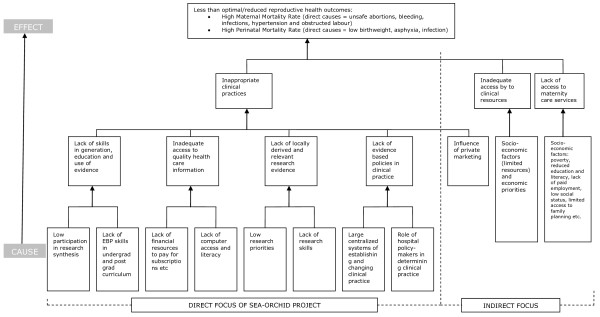
Project rationale: problem analysis

The starting point for the problem analysis (Figure 3) was the less than optimal reproductive health outcomes in South East Asia, as reflected by high maternal and perinatal mortality. The direct causes of maternal mortality were understood to be unsafe abortions, bleeding, infections, hypertension and obstructed labour; and of perinatal mortality were understood to be low birth weight, asphyxia and infection. In response to this, the overarching goal developed for the SEA-ORCHID project was '*To improve the health of mothers and babies in South East Asia*'.

Three issues were identified as underlying the central problem of poor reproductive outcomes. These were:

1. Inappropriate clinical practices

2. Inadequate access to clinical resources

3. Lack of access to maternity care services

Since SEA-ORCHID was not a multi-million dollar international aid project, our focus was on the first of these. The purpose of the SEA-ORCHID project was thus defined as '*To improve clinical practice in reproductive health in South East Asia*'.

The problem analysis outlined several reasons why clinical practice was not always in line with the results of research. These included a lack of skills in generating and using evidence; inadequate access to reliable healthcare information; lack of locally derived and relevant research evidence; and lack of evidence-based policies and clinical practice guidelines (Figure 3).

These reasons were determined to have several underlying causes:

• low participation in research synthesis

• lack of evidence-based practice training in medical and nursing curricula

• lack of financial resources to pay for subscriptions, etc

• lack of computer access and literacy

• low priority given to research

• lack of research skills

• large centralized systems of establishing and changing clinical practice

• role of hospital policy-makers in determining clinical practice.

### Project Planning

**Figure 4:**
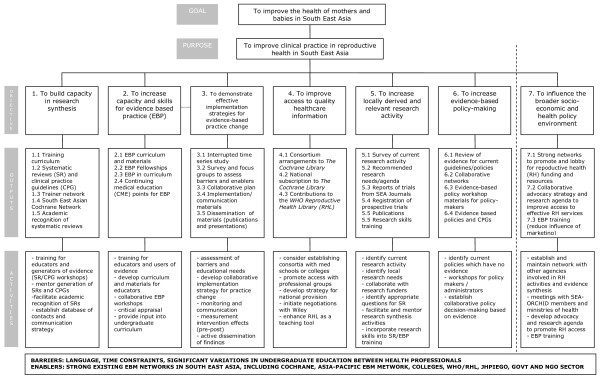
Project description

In the project planning phase, project objectives were developed to address these underlying causes (Figure 4). The objectives of the SEA-ORCHID project were:

1. to build capacity in research synthesis

2. to increase capacity and skills for evidence-based practice

3. to demonstrate effective implementation strategies for evidence-based practice change

4. to improve access to quality healthcare information

5. to increase locally derived and relevant research activity

6. to increase evidence-based policy making

7. to influence the broader socio-economic and health policy environment.

In relation to the first objective '*To build capacity in research synthesis*', proposed activities included training in systematic reviews and guideline development, developing structures to sustain research synthesis activity in the longer term, and facilitating recognition of systematic reviews as a valid form of academic research.

Activities linked to the second objective '*To increase capacity and skills for evidence-based practise' *included developing curriculum materials, training educators and users of evidence, and working towards the integration of EBP within existing curricula.

In relation to the third objective *'To demonstrate effective implementation strategies for evidence-based practice change*', activities focused on assessing barriers and educational needs, promulgating adult learning principles, monitoring clinical practice and disseminating findings, and developing collaborative implementation strategies.

The fourth objective *'To improve access to quality healthcare information' *gave rise to activities that included pursuing consortia with medical schools or colleges, promoting access to various professional groups, and developing a longer term strategy to secure national provision to *The Cochrane Library*.

The fifth objective *'To increase locally derived and relevant research activity' *included activities aimed at identifying local research needs and appropriate questions for systematic reviews, facilitating and mentoring those involved in research synthesis, and incorporating research skills into training programs.

Activities in relation to objective six *'To increase evidence-based policy making' *included identifying evidence gaps and organising workshops for policy makers.

The final objective *'To influence the broader socio-economic and health policy environment' *was concerned with activities around advocacy and awareness raising, and collaborating with other agencies.

These broad objectives and activity descriptions were used by the project teams in each country and site to develop and implement detailed project plans reflecting local priorities, needs and resources. Descriptions of the specific activities undertaken at each participating hospital are available at http://www.seaorchid.org.

### Evaluation and measurement

#### Outcome Measures

**Figure 5:**
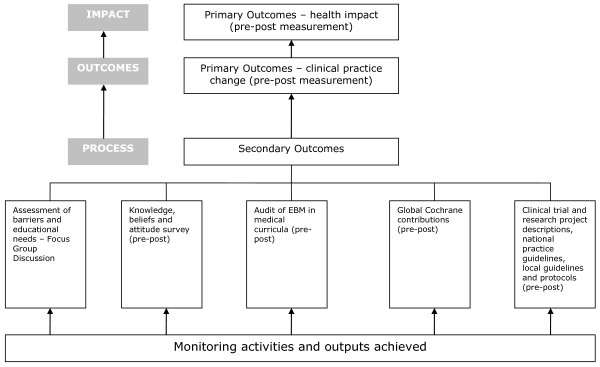
Project evaluation and measurement

To establish whether the project met its goal of improving health for mothers and babies and its purpose of improving perinatal clinical practice, primary outcome measures were designed to evaluate change in maternal and neonatal health outcomes and 15 key evidence-based clinical practices. At each of the participating hospitals we collected data on 1000 births before and after the project intervention to determine changes in health outcomes and practices (Figure 5). Specific outcome measures were also established to evaluate each of the project objectives. These secondary outcome measures are provided in Table 2.

**Table 2:** Objectives and related outcome measures of the SEA-ORCHID Project

Project Objective	Outcome measures
To build capacity in research synthesis	SR and CPG workshops conducted
	SRs and CPGs produced
	SR trainers on database
	Participants in Cochrane Collaboration
	Branches of Australasian Cochrane Centre in SE Asia
	Academic sites recognising SRs in SE Asia

To increase capacity and skills for evidence-based practice	Training materials developed
	EBP sessions undertaken and number of participants
	EBP fellowships
	EBP in curricula pre- and post-intervention
	Nodes with documented recognition of CME points
	Persons receiving CME points for EBP training

To demonstrate effective implementation strategies for evidence-based practice change	Pre- and post-intervention measurement of primary and secondary outcomes
	Qualitative assessment of barriers and enablers to EBP
	Knowledge, beliefs and attitudes to EBP
	Program logic framework with input and consensus from all project partners
	Process evaluation of project implementation
	Description of collaboration and communication strategies
	Publication describing current practice

To improve access to quality healthcare information	*Cochrane Library *usage statistics
	National subscriptions to *Cochrane Library*
	RHL contributions

To increase locally derived and relevant research activity	Research activities
	Reports of trials identified and submitted to the Cochrane Central Register of Controlled Trials
	Research trials registered
	Papers published
	Training sessions conducted
	Participants attending training sessions

To increase evidence-based policy making	Description of networking arrangements and communication strategy and members
	Collaborative activities
	Workshops conducted
	Participants attending workshops
	EB policies and CPGs

To influence the broader socio-economic and health policy environment	Documented advocacy strategies
	Advocacy and lobbying networks and activities
	Responses and outcomes to advocacy activities

Systematic Review (SR), Clinical Practice Guidelines (CPG), Evidence-Based Practice (EBP), Continuing Medical Education (CME), WHO Reproductive Health Library (RHL)

#### Process measures

Assessment of the progress of the project was done through face-to-face project meetings, teleconferences and regular reporting. Project meetings involving team members from all SEA-ORCHID sites were held every nine months to provide an opportunity for sharing successes, brainstorming approaches to overcoming barriers, encouraging networking across nodes and refining the planned activities for the next period of the intervention phase. Between project meetings, the Australian educators initiated regular two-monthly teleconferences with each of the project nodes to ensure progress was on track, provide support and resources, and help with any problems or issues that arose. The project investigators also had regular teleconferences. SEA-ORCHID team members reported every two months on the activities undertaken at their hospital by completing activity statements which were then uploaded to the SEA-ORCHID website http://www.seaorchid.org to give a cumulative summary of all activities undertaken during the project.

## Discussion

There are many different program logic models and frameworks used for planning and evaluating programs. Despite differences in approach, they share a common purpose which is to describe the rationale behind a program and to clarify the relationships between monitoring and evaluation and the project activities. In development projects, logical frameworks are the predominant program logic method used and are particularly important as communication tools in projects that cover diverse countries, cultures and languages [16]. The purpose of using a logical framework in the SEA-ORCHID project was to:

• assess whether the project activities were addressing perceived needs;

• clarify the expected cause-and-effect relationships between the project activities and expected outcomes;

• predict the extent to which the planned outcome measures were likely to evaluate the impact of the project activities; and

• assist project workers to reach a consensus about realistic goals and outcomes of the project.

The logical framework became the core tool that guided the overall intervention strategy and was taken by each site and used as the basis for devising a tailored suite of activities that was implemented within an action research framework during the two-year intervention phase (2006-07). Over the course of the project the logical framework was a powerful tool for facilitating communication within and between sites, and for maintaining a shared understanding of the purpose and progress of a complicated, multilayered intervention.

There are several limitations of the logical framework approach for projects of this nature. Firstly, the linear model does not always do justice to the complex multifaceted influences, dynamic interactions, cultural differences and unforeseen consequences that are part of everyday reality. Therefore they need to be used flexibly and adapted as projects evolve. Secondly, the logical framework only represents the consensus of the individuals involved in developing it. However, they can be used to clarify the assumptions on which project plans are made and allow incoming members of the project to check whether these assumptions are relevant to their local context. Finally, the logical framework approach in this instance had to be adapted to meet the realities of planning a project intervention within the constraints of a research grant. Under ideal conditions, projects are planned systematically by looking first at the problem, then developing goals and objectives, and finally devising an accompanying evaluation plan [17]. In SEA-ORCHID, the broad issue of building capacity for evidence-based practice and the pre- and post-intervention evaluation strategy was defined first and the specific goals and objectives defined subsequently within the requirements of a research protocol.

Experts in healthcare improvement emphasise the crucial importance of acquiring a good understanding of the problem, the target group, its setting and obstacles to change [5]. Logic models, such as the logical framework, value participatory processes in project planning as a means of achieving this understanding. SEA-ORCHID is an international collaborative project that aims to improve health outcomes for mothers and babies in South East Asia by strengthening capacity for generating, synthesising and implementing evidence. The logical framework approach is an established project design method that is based on a systematic analysis of the problem or situation and an exploration of possible solutions. For SEA-ORCHID, the use of a project-wide logical framework has provided a mechanism for further developing site-specific intervention plans. Overall, this approach is both robust and flexible and appropriate for real-world research into change processes.

## Conclusions

The process of developing a logical framework in the SEA-ORCHID project was valuable because it enabled a reasoned, logical approach to the project design that ensured the project activities would achieve the desired outcomes and that the evaluation plan would assess both the process and outcome of the project. The logical framework was also valuable over the course of the project to facilitate communication, provide a reference for assessment of progress and build a shared understanding of the project activities, purpose and goal.

## Abbreviations

EBP: Evidence-based practice; SE Asia: South East Asia; SEA-ORCHID: South East Asia - Optimising Reproductive and Child Health in, Developing Countries; WHO: World Health Organization

## Competing interests

The authors declare that they have no competing interests.

## Authors' contributions

DH-S, PL, SG and CAC conceived the SEA-ORCHID study. SM and CC devised and refined the logical framework based on contributions from the investigators. SM wrote the initial draft of the manuscript which TT revised. All investigators and Australian educators were involved in developing the design of the project, critically revising the manuscript for intellectual content and have given final approval of the version to be published.

## Pre-publication history

The pre-publication history for this paper can be accessed here:

http://www.biomedcentral.com/1471-2288/10/61/prepub

## Supplementary Material

Additional file 1 **SEA-ORCHID survey**. Click here for file
