# Supplementary material for: Building capacity for evidence generation, synthesis and implementation to improve the care of mothers and babies in South East Asia: methods and design of the SEA-ORCHID Project using a logical framework approach
Source: BMC Med Res Methodol. 2010 Jul 1;10:61. doi: 10.1186/1471-2288-10-61 (PMC2912918; doi:10.1186/1471-2288-10-61)
Supplement: Additional file 1 — SEA-ORCHID survey. [file 1471-2288-10-61-S1.DOC]

# Current practice information from SE Asia centres

| SEA-ORCHID site: |  |
| --- | --- |

# 1. Use of evidence-based approach to care in your area

To help determine which setting(s)/hospitals we should be targeting for greatest impact, please indicate how widespread the use of an evidence-based approach to care is for each of the settings below. [Tick or mark ‘X’ in the most appropriate box]

## Tertiary (teaching) hospital

|  | Always | Commonly | Uncommonly | Rarely |
| --- | --- | --- | --- | --- |
| Clinicians |  |  |  |  |
| Policy makers |  |  |  |  |
| Undergraduate education |  |  |  |  |
| Midwifery/nursing |  |  |  |  |

## Regional hospital

|  | Always | Commonly | Uncommonly | Rarely |
| --- | --- | --- | --- | --- |
| Clinicians |  |  |  |  |
| Policy makers |  |  |  |  |
| Undergraduate education |  |  |  |  |
| Midwifery/nursing |  |  |  |  |

## Smaller district hospital

|  | Always | Commonly | Uncommonly | Rarely |
| --- | --- | --- | --- | --- |
| Clinicians |  |  |  |  |
| Policy makers |  |  |  |  |
| Undergraduate education |  |  |  |  |
| Midwifery/nursing |  |  |  |  |

Any further comments about which setting(s) or hospitals we should be targeting for greatest impact?

|  |
| --- |

Are there factors we need to be aware of, such as known local barriers to an evidence-based approach or practice change?

|  |
| --- |

Are you or is your institution involved in the *WHO Reproductive Health Library* cluster randomised trial?

|  |
| --- |

To what extent are protocols/guidelines part of clinical care and the implementation of research?

|  |
| --- |

**2. Current research in your setting**

Please list any research underway that may inform us about maternity and neonatal care, e.g. audits, cohorts, RCTs etc.?

|  |
| --- |

Is there any ongoing research that may be affected by this project?

|  |
| --- |

Are there staff already trained for data collection?

|  |
| --- |

**3. Project interventions and outcomes**

# Are there existing data collections in your region for maternal and child health? If yes, please describe them.

|  |
| --- |

For the areas of practice listed in the table below we want to identify how relevant the interventions are to your setting and the extent to which they are routinely used in standard practice. Please complete the table using a 1-10 Likert scale.

Table of interventions

**Antenatal practices**

| Intervention | Relevance  1=not relevant at all 10=very relevant | | Standard practice  1=never used  10=used routinely all the time | |
| --- | --- | --- | --- | --- |
|  | Tertiary (teaching) hospital | Regional hospital | Tertiary (teaching) hospital | Regional hospital |
| Use of magnesium sulphate for women with eclampsia and pre-eclampsia |  |  |  |  |
| Use of corticosteroids prior to pre-term birth where presentation is <34 weeks |  |  |  |  |
| ECV at term |  |  |  |  |

**Intrapartum interventions**

| Continuous support for women in labour |  |  |  |  |
| --- | --- | --- | --- | --- |
| Selective use of episiotomy |  |  |  |  |
| Active management of third stage labour |  |  |  |  |
| Vacuum rather than forceps for operative delivery |  |  |  |  |
| Intra-operative antibiotics at time of caesarean section |  |  |  |  |
| Interventions to reduce HIV transmissions during birth (use of anti-retroviral agents and/or caesarean section) |  |  |  |  |

**Postnatal interventions**

| Unrestricted infant access for breast feeding |  |  |  |  |
| --- | --- | --- | --- | --- |
| Antiseptic umbilical cord care |  |  |  |  |
| Intubation at resuscitation of infant with meconium stained liquor |  |  |  |  |
| Hepatitis B immune globulin at birth |  |  |  |  |
| Kangaroo care |  |  |  |  |

Are there other health interventions that have systematic review evidence for that would be worth adding?

|  |
| --- |

Do you have data available to inform sample size calculations for the interventions and outcomes in the table below? Please comment below or complete the table.

|  |
| --- |

Can you provide data on the current rates of use of each of the interventions at your centre and/or other hospitals within your region, even if approximate. State source of data if possible. Please complete the table below.

**Antenatal practices**

| **Intervention** | Current rates of use  (percent) | | Outcomes | Data available?  mark Yes or No | |
| --- | --- | --- | --- | --- | --- |
|  | Tertiary hospial | Regional hospital |  | Tertiary hospital | Region hospital |
| Use of magnesium sulphate for women with eclampsia and pre-eclampsia |  |  | Maternal death  Eclampsia |  |  |
| Use of corticosteroids prior to pre-term birth where presentation is <34 weeks |  |  | Neonatal death |  |  |
| ECV at term |  |  | Caesarean section for breech at term |  |  |

**Intrapartum interventions**

| Continuous support for women in labour |  |  | Caesarean section rate |  |  |
| --- | --- | --- | --- | --- | --- |
| Selective use of episiotomy |  |  | Maternal perineal injury  Maternal infection |  |  |
| Active management for third stage labour |  |  | Major postpartum hemorrhage  Maternal death |  |  |
| Vacuum rather than forceps for operative delivery |  |  | Maternal perineal injury |  |  |
| Intra-operative antibiotics at time of caesarean section |  |  | Maternal infection |  |  |
| Interventions to reduce HIV transmissions during birth (use of anti-retroviral agents and/or caesarean section) |  |  | HIV positive infants |  |  |

**Postnatal interventions**

| Unrestricted infant access for breast feeding |  |  | Formula feeding  Infant infection |  |  |
| --- | --- | --- | --- | --- | --- |
| Anticeptic umbilcal cord care |  |  | Umbilical and infant infection |  |  |
| Intubation at resuscitation of infant with meconium stained liquor |  |  | Admission to neonatal intensive care |  |  |
| Hepatitis B immune globulin at birth |  |  | Was the immunization given? |  |  |
| Kangaroo care |  |  | Reduced neonatal morbidity  Breast feeding rate |  |  |
